# Preclinical Evaluation of the Pan-FGFR Inhibitor LY2874455 in FRS2-Amplified Liposarcoma

**DOI:** 10.3390/cells8020189

**Published:** 2019-02-21

**Authors:** Robert Hanes, Else Munthe, Iwona Grad, Jianhua Han, Ida Karlsen, Emmet McCormack, Leonardo A. Meza-Zepeda, Eva Wessel Stratford, Ola Myklebost

**Affiliations:** 1 Department of Tumor Biology, Institute of Cancer Research, the Norwegian Radium Hospital, Oslo University Hospital, 0379 Oslo, Norway; Robert.Hanes@rr-research.no (R.H.); Else.Munthe@rr-research.no (E.M.); Iwona.Grad@rr-research.no (I.G.); Leonardo.A.Meza-Zepeda@rr-research.no (L.A.M.-Z.); eva.wessel.stratford@rr-research.no (E.W.S.); 2 Norwegian Cancer Genomics Consortium, 0379 Oslo, Norway; 3 Centre for Cancer Biomarkers (CCBIO), Department of Clinical Sciences, University of Bergen, 5021 Bergen, Norway; Jianhua.Han@uib.no (J.H.); idakarlsenemail@gmail.com (I.K.); emmet.mc.cormack@med.uib.no (E.M.); 4 KinN Therapeutics AS, 5021 Bergen, Norway; 5 Department of Internal Medicine, Hematology Section, Haukeland University Hospital, 5021 Bergen, Norway; 6 Genomics Core Facility, Department of Core Facilities, Institute of Cancer Research, the Norwegian Radium Hospital, Oslo University Hospital, 0379 Oslo, Norway; 7 Department of Clinical Science, University of Bergen, 5020 Bergen, Norway

**Keywords:** FRS2, FGFR, NVP-BGJ398, LY2874455, sarcoma

## Abstract

**Background:** FGFR inhibition has been proposed as treatment for dedifferentiated liposarcoma (DDLPS) with amplified *FRS2*, but we previously only demonstrated transient cytostatic effects when treating *FRS2*-amplified DDLPS cells with NVP-BGJ398. **Methods:** Effects of the more potent FGFR inhibitor LY2874455 were investigated in three DDLPS cell lines by measuring effects on cell growth and apoptosis *in vitro* and also testing efficacy *in vivo*. Genome, transcriptome and protein analyses were performed to characterize the signaling components in the FGFR pathway. **Results:** LY2874455 induced a stronger, longer-lasting growth inhibitory effect and moderate level of apoptosis for two cell lines. The third cell line, did not respond to FGFR inhibition, suggesting that *FRS2* amplification alone is not sufficient to predict response. Importantly, efficacy of LY2874455 was confirmed *in vivo*, using an independent *FRS2*-amplified DDLPS xenograft model. Expression of *FRS2* was similar in the responding and non-responding cell lines and we could not find any major difference in downstream FGFR signaling. The only FGF expressed by unstimulated non-responding cells was the intracellular ligand FGF11, whereas the responding cell lines expressed extracellular ligand FGF2. **Conclusion:** Our study supports LY2874455 as a better therapy than NVP-BGJ398 for *FRS2*-amplified liposarcoma, and a clinical trial is warranted.

## 1. Introduction

Sarcomas are rare cancers of mesenchymal origin, accounting for approximately 1% of all solid cancers, and can be classified into more than 50 distinct histological subtypes [1]. Liposarcomas (LPS), which resemble adipose tissue, are further classified into three main subtypes, well-differentiated/dedifferentiated liposarcoma (WD/DDLPS), myxoid/round cell liposarcoma, and pleomorphic liposarcoma [2]. The heterogeneity makes clinical research and trials challenging. However, all together rare cancers comprise one of the largest patient groups, which is in great need of new therapeutic approaches. Therefore, a deeper mechanistic understanding is needed to be able to identify and validate new potential targets. In a previous study, we identified amplifications of multiple genes in the 12q14.1-q15 region in the DDLPS cell line NRH-LS1 and investigated several of these as therapeutic targets [3]. One of these, *FRS2*, is generally co-amplified with *MDM2* in WD/DDLPS [4]. *FRS2* codes for an important component of the FGF receptor (FGFR) signaling pathway, which plays crucial roles in multiple biological processes, such as cell growth, survival and differentiation, as well as tumor development and progression [5,6]. FRS2-dependent FGFR signaling is induced through FGFR activation by FGF ligands, and consecutive phosphorylation of FRS2 triggers an intracellular signaling cascade involving RAS/MAPK/ERK and PI3K/AKT [7], leading to oncogenic pro-survival and anti-apoptotic properties and increased proliferation and migration. To date, there are no drugs available that can target FRS2 directly, but attenuating the signal from FGFR, upstream of FRS2, with FGFR inhibitors has been shown to be growth inhibitory in such cells [3,8].

NVP-BGJ398, which is in phase II clinical trials, has been shown to be a potent and selective FGFR inhibitor in a wide panel of cancer cell lines [9]. NVP-BGJ398 has been reported to selectively inhibit FGFR1, –2 and –3 with IC50s of 0.9 nM, 1.4 nM and 1.0 nM, respectively, whereas the IC50 for FGFR4 is 60 nM [10]. Another pan-FGFR inhibitor, LY2874455, recently completed a phase I clinical trial [11], and has been reported to selectively inhibit FGFR1, –2, –3, and –4 with IC50s of 2.8 nM, 2.6 nM, 6.4 nM and 6 nM, respectively [12].

In this study, we have investigated the therapeutic potential of LY2874455 with the aim to improve efficacy for *FRS2*-amplified DDLPS.

## 2. Materials and Methods

### 2.1. Cell Line and Culture Conditions

The DDLPS cell lines NRH-LS1, established from a patient-derived xenograft as previously described [3] and LPS510 and LPS853, kindly provided by Dr. Jonathan Fletcher, were cultured in RPMI-1640 medium (Sigma-Aldrich, St. Louis, MO, USA) supplemented with 10% FBS (Sigma-Aldrich), 1% l-alanyl-l-glutamine (Sigma-Aldrich) and 1% penicillin-streptomycin (Sigma-Aldrich) and grown at 37 °C, 5% CO_2_. Short tandem repeat DNA profiling was performed on all cell lines to confirm identity. Cells were negative for mycoplasma using the VenorGeM Mycoplasma Detection Kit (Minerva Biolabs, Berlin, Germany).

### 2.2. Drugs

LY2874455 (#S7057) (Selleck Chemicals, Munich, Germany) was dissolved in DMSO (Sigma-Aldrich) according to the manufacturer’s recommendation. For each experiment the appropriate control (referred to as untreated) was used, with a DMSO concentration corresponding to that used with the highest drug concentration. The concentration of DMSO in the control for 100 nM LY2874455 and NVP-BGJ398 was 0.01% and for 1 μM LY2874455 and NVP-BGJ398 0.1%.

### 2.3. Drug Treatment and Cell Proliferation Assay

The cellular proliferation rate was measured using a live-cell imaging system, IncuCyte ZOOM (Essen Bioscience, Birmingham, UK) with the corresponding software application (version 2013BRev1), (Essen Bioscience, Birmingham, UK). Cells were seeded into 96-well plates, and drug treatment initiated after 16h, in triplicates. Each drug treatment was performed over a period of 1–2 weeks and done at least three times. Proliferation rate was measured as cell confluence over time every third hour.

### 2.4. Apoptosis

Apoptosis assays were done with the IncuCyte ZOOM. To measure apoptosis, the CellPlayer 96-Well Kinetic Caspase-3/7 reagent containing DEVD-NucViewTM488 (Essen Bioscience) at a concentration of 2 μM was added at the same time as the drug. Total numbers of apoptotic cells were counted in the green channel (488 nm). After 96 h, cells were incubated for 30 minutes with 4 µM of Nuclear-ID Red DNA stain (Enzo Life Sciences, Farmingdale, NY, USA), and total cell count was measured in the red channel (566 nm). The percentage of apoptotic cells per well was calculated as the number of apoptotic cells relative to the total number of nuclei.

### 2.5. Viability Assay for Dose-Response Curve and IC50 Calculations

Cell viability was measured using the CellTiter-Glo Luminescent Cell Viability Assay (Promega, Madison, WI, USA). 5 × 10^3^ cells per well were seeded onto a 96-well flat and clear bottom polystyrene treated microplate (Corning, Corning, NY, USA). The drug treatment was initiated 16 h after seeding and was applied at concentrations ranging from 0.1 nM to 1000 nM. After 72, 96 and 120 h, ATP levels were used as a measure of viability. Relative IC50 for 120 h was calculated using a four-parameter logistic function [13] based on non-linear regression analysis using SigmaPlot (Systat Software Inc, San Jose, CA, USA) version 12.5.0.38.

### 2.6. In Vivo Assay

Animal experiments were performed according to protocols approved by the National Animal Research Authority (Mattilsynet) in compliance with the European Convention of the Protection of Vertebrates Used for Scientific Purposes (ID 10175). The LS70x xenograft (established directly from a DDLPS tumor) was implanted into the flank of immunodeficient NOD-scid IL2rγnull (NSG) mice [14]. When tumors reached 150 mm^3^, animals were randomized into two groups, each of six mice, and treated twice per day with either 3 mg/kg LY2874455 or vehicle only (2% (*v*/*v*) DMSO, 30% (*v*/*v*) PEG 300, 5% (*v*/*v*) Tween 80 in sterile water), administered by oral gavage. Treatment was performed for 28 days, or until the tumor reached a size of 1 cm^3^. Tumor growth was measured by caliper measurements twice per week for the duration of treatment. Unpaired two-tailed *t*-test was performed to detect significant differences in tumor volumes (*p* ≤ 0.05 was considered significant).

### 2.7. Western Blots

Cells were treated for 24 h with either 100 nM LY2874455, 100 nM NVP-BGJ398 or control-treated with the corresponding concentration of DMSO and the last 15 min with or without 15 ng/ml of recombinant human FGF1 [15] and 10 U/mL of Heparin. *In vitro* cells were washed with PBS and dissolved in SDS lysis buffer. Xenografts were cut into smaller pieces and snap frozen. Proteins were extracted with T-Per lysis buffer (Thermo Fisher Scientific, Waltham, MA, USA), supplemented with protease and phosphatase inhibitors (both from Thermo Fischer Scientific), using the TissueLyser LT (QIAGEN, Venlo, Netherlands). DTT was added to the lysates before boiling. Proteins were separated in a 4–12% Novex PAGE gel in MOPS running buffer, and transferred to PVDF membranes (Thermo Fisher Scientific). The following antibodies were used: pFRS2-TYR436 (#3861), AKT (#9272), pAKT-SER473 (#9271), ERK (#9102), pERK-T202/Y204 (#4370), PLCγ1 (#5690), pPLCγ1-TYR783 (#2821) (all from Cell Signaling Technology, Danvers, MA, USA), FRS2 (#SC8318) (Santa Cruz Biotechnology, Dallas, TX, USA) and α-Tubulin (#CP06) (Merck KGaA, Darmstadt, Germany). All antibodies were diluted 1:1000, except FRS2 (1:500) and α-Tubulin (1:2000). Secondary antibodies were rabbit anti-mouse immunoglobulins/HRP (#P0260) and goat anti-rabbit immunoglobulins/HRP (#P0448) (Dako, Glostrup, Denmark) at a concentration of 1.3 g/L and 0.25 g/L respectively. The Western blots were developed using the Supersignal Western Dura substrate (Thermo Fisher Scientific), and detected and quantified on a Syngene G-Box (Synoptics Group, Cambridge, UK) with the GeneSnap (version 7.12, Synoptics Group) and the GeneTools (version 4.3.7.0, Synoptics Group) programs, respectively.

### 2.8. Quantitative Real-Time PCR-Based Copy Number Assay

DNA was isolated from cells using the AllPrep DNA/RNA Mini Kit (QIAGEN) according to the manufacturer’s protocol. Quantitative real-time PCR was performed based on absolute quantitation using the Applied Biosystems 7900HT fast real-time PCR system (Applied Biosystems, Foster By, CA, USA). The copy numbers of *FRS2* (Hs02860563_cn), *ALB* (Hs05929625_cn) and *LSAMP* (Hs05902664_cn) were determined using TaqMan copy number assays from Applied Biosystems, *ALB* and *LSAMP* were used as endogenous controls, as these have low level of DNA copy number changes in a large panel of liposarcoma samples [16]. The copy numbers were determined using the CopyCaller Software v2.1 program (Applied Biosystems) as described by the manufacturer, and the FRS2 data were normalized to *LSAMP*. The copy numbers were validated using *ALB* as another endogenous reference gene (data not shown).

### 2.9. Quantitative Real-Time PCR Based Expression Assay

RNA was isolated from cells using the AllPrep DNA/RNA Mini Kit (QIAGEN) according to the manufacturers protocol. cDNA was prepared using 1 µg of RNA and the SuperScript VILO Master Mix (Invitrogen, Carlsbad, CA, USA). Quantitative real-time PCR was performed based on ΔΔCt relative quantitation using the Applied Biosystems 7900HT fast real-time PCR system (Applied Biosystems). The expression levels of FRS2 were determined using TaqMan gene expression assays (Hs00183614_m1) with human B2M (VIC^®^/MGB probe) (Applied Biosystems) as internal control for normalization. The relative expression levels were determined using the comparative ΔΔCt method as described by the manufacturer. Human Adipose Tissue Total RNA was used as reference (Clontech, Mountain View, CA, USA).

### 2.10. RNA Sequencing

RNA was isolated from the cell lines using the AllPrep DNA/RNA Mini Kit (QIAGEN). mRNA sequencing libraries were prepared using 100 ng of total RNA and the Illumina TruSeq Stranded mRNA Library Prep kit for NeoPrep following the supplier’s instructions. The libraries were sequenced on a NextSeq 500 Illumina sequencer (Illumina, San Diego, CA, USA) using a High Output v2 kit chemistry, generating 2 × 75 bp paired-end sequence reads. RNA-Seq reads were aligned using STAR aligner (v.2.5.0b) against the human reference genome (UCSC hg19, RefSeq and Gencode gene annotations), and FPKM estimation was generated by Cufflinks 2 using the RNA-seq alignment app at Illumina BaseSpace.

## 3. Results

### 3.1. Improved Efficacy Using LY2874455

**Figure 1:**
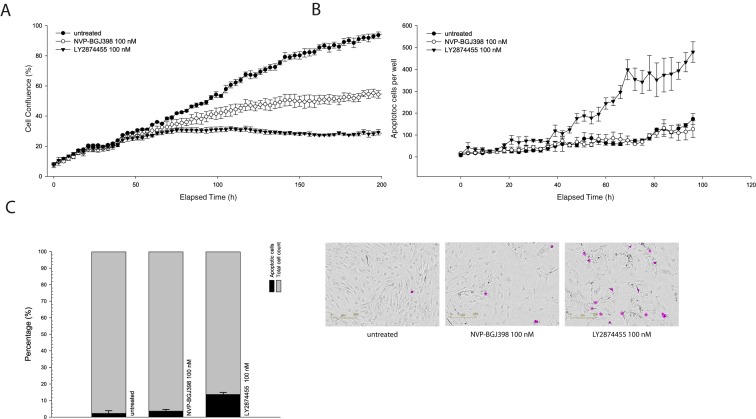
Comparison of the effect of NVP-BGJ398 and LY2874455 on proliferation and apoptosis of NRH-LS1 cells. (**A**) Proliferation of NRH-LS1 cells after inhibition of FGFR with either NVP-BGJ398 or LY2874455; one representative experiment is shown (*n* = 3), error bars represent the standard error of the mean (SEM) of technical replicates; (**B**) The number of cells with active caspase 3/7 during 96 h of treatment with either 100 nM of NVP-BGJ398 or 100 nM LY2874455, one representative experiment is shown (*n* = 3); (**C**) The percentage of apoptotic cells after 96 h of treatment with NVP-BGJ398 or LY2874455; the mean of experiments is shown (*n* = 3), error bars represent the standard deviation (SD) of the experiments. Representative images show apoptotic cells outlined in purple based on measured apoptotic signal. For all experiments untreated is with DMSO concentration corresponding to that of the highest drug concentration.

**Figure 2:**
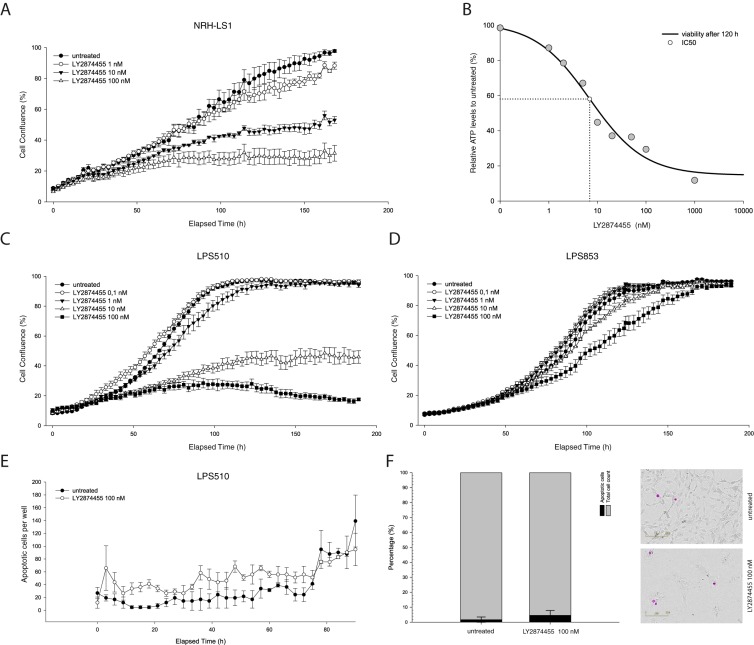
The effect of LY2874455 on the proliferation and viability of NRH-LS1, LPS510 and LPS853 cells. (**A**) Proliferation of NRH-LS1 cells at different concentrations of LY2874455; one representative experiment is shown (*n* = 3), error bars represent the standard error of the mean (SEM) of technical replicates; (**B**) The IC50 was estimated at 7 nM based on NRH-LS1 cell viability after 120 h of treatment with LY2874455; Proliferation of LPS510 (**C**) and LPS853 (**D**) cells in the presence of LY2874455. (**E**) The number of LPS510 cells with active caspase 3/7 during 96 h of treatment with 100 nM of LY2874455; (**C**–**E**) One representative experiment is shown (*n* = 3), error bars represent the standard error of the mean (SEM) of technical replicates; (**F**) The percentage of apoptotic LPS510 cells after 96 h of treatment with 100 nM of LY2874455. The mean of experiments is shown (*n* = 4), error bars represent the standard deviation (SD) of the experiments. Representative images show apoptotic cells outlined in purple based on measured apoptotic signal. For all experiments untreated is with DMSO concentration corresponding to that of the highest drug concentration.

When treating NRH-LS1 cells at equivalent concentrations, LY2874455 inhibited growth of the cells more efficiently than did NVP-BGJ398 (Figure 1A). NRH-LS1 cells exposed to 100 nM of LY2874455 were completely growth inhibited after 72 h (Figure 1A), while treatment with 100 nM NVP-BGJ398 gave only partial growth inhibition at that time point. In contrast to NVP-BGJ398, we found that LY2874455 induced apoptosis in a subpopulation of NRH-LS1 cells (Figure 1B). Furthermore, 100 nM LY2874455 induced, on average, four times higher levels of apoptosis after 96 h, as compared to cells treated with 100 nM NVP-BGJ398 (Figure 1C). As shown in Figure 2, LY2874455 inhibited cell growth in a dose-dependent manner (Figure 2A), with the full effect at approximately 100 nM. The IC50 for LY2874455 in NRH-LS1 cells was estimated to 7 nM compared to 47 nM for NVP-BGJ398 (Figure S2), based on viability after 120 h of treatment (Figure 2B).

### 3.2. The Response to FGFR Inhibition in FRS2-Amplified Cell Lines Is Variable

We next investigated the effect of LY2874455 in two additional *FRS2*-amplified DDLPS cell lines, LPS510 and LPS853, which have *FRS2* copy number and gene expression levels comparable to NRH-LS1 (Figure S1). The expression levels of FRS2 were 10–20 fold higher in all three cell lines compared to both human adipocyte tissue and an undifferentiated immortalized mesenchymal progenitor cell line (iMSC#3), [17] (Figure S1). LY2874455 inhibited the growth of LPS510 similar to NRH-LS1 (Figure 2C), with IC50 values of 5.5 and 6.9 respectively (Figure S2). In contrast, LPS853 was only modestly inhibited at 100 nM (Figure 2D). Similar levels of apoptosis were observed for both LPS510 and NRH-LS1 after treatment with 100 nM LY2874455, with 9% for LPS510 cells (Figure 2E,F) and 13% for NRH-LS1 (Figure 1C) at 96 h.

### 3.3. LY2874455 Induces Long-Lasting Growth Inhibition

**Figure 3:**
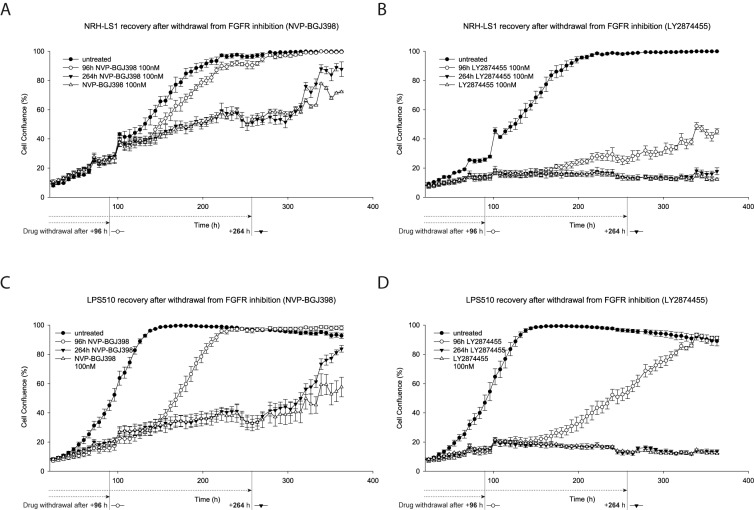
NRH-LS1 and LPS510 cells resume proliferation after withdrawal of treatment with NVP-BGJ398, but not LY2874455. Proliferation of NRH-LS1 (**A**,**B**) or LPS510 (**C**,**D**) cells treated with 100 nM of NVP-BGJ398 (**A**,**C**) or LY2874455 (**B**,**D**) continuously or upon withdrawal of the drug after 96 h or 264 h of treatment. One representative experiment is shown (*n* = 3), error bars represent the standard error of the mean (SEM) of technical replicates. For all experiments untreated with DMSO concentration corresponding to that of the highest drug concentration.

In order to assess duration of the growth inhibitory effects of LY2874455 and NVP-BGJ398, we discontinued drug treatment after 96 h or 264 h. In contrast to NVP-BGJ398, the growth inhibition of NRH-LS1 and LPS510 was strong after withdrawal of LY2874455, although it was quite significant with LPS510 cells, especially at the highest doses (Figure 3A–D). Both LPS510 and NRH-LS1 maintained growth arrest within the time-frame of the experiment after 264 h of treatment with LY2874455.

### 3.4. Both FGFR Inhibitors Inactivate MEK/ERK-Dependent Signaling in FRS2-Amplified Cells

**Figure 4:**
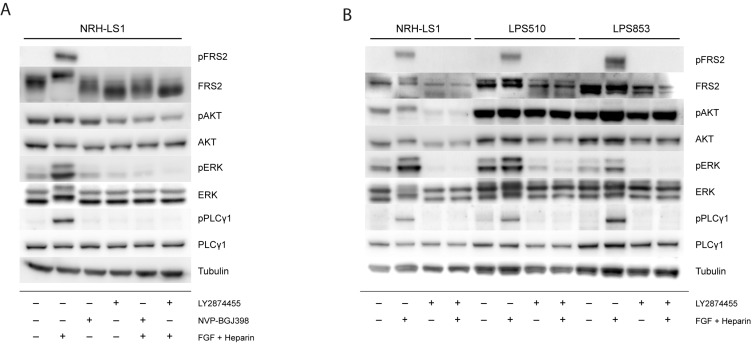
Signaling pathway analysis after FGFR stimulation and inhibition in NRH-LS1, LPS510 and LPS853. Western blots showing (**A**) The level of phosphorylated and total protein for the indicated proteins in NRH-LS1 cells treated for 24 h with 100 nM of LY2874455 or 100 nM NVP-BGJ398, with or without FGF stimulation as indicated; (**B**) The level of phosphorylated and total protein for the indicated proteins in NRH-LS1, LPS510 and LPS853 cells treated for 24 h with 100 nM of LY2874455, with or without FGF as indicated. In all Western blot experiments α-tubulin was used as a loading control.

To determine whether the different effects of these drugs could be attributed to the activation or inhibition of different signaling components in the FGFR pathway, we investigated the status of several signaling proteins downstream of FGFR and FRS2 in NRH-LS1 cells upon stimulation with FGF and treatment with NVP-BGJ398 or LY2874455 (Figure 4, quantified in Figure S4A). Although phosphorylation of FRS2 was expected to drive growth in these cells, we only detected phosphorylated FRS2 upon stimulation with exogeneous FGF, probably because pFRS2 levels in unstimulated cells were below the detection limit of the pFRS2 antibody used in this western blot assay. An increased phosphorylation of the downstream signaling protein ERK was also found upon stimulation with FGF1. When NRH-LS1 cells were treated for 24 h with either 100 nM LY2874455 or 100 nM NVP-BGJ398, FGF1-induced phosphorylation of FRS2 was completely abolished (Figure 4A), supporting the expected drug action, and also endogenous pERK was reduced. Stimulation with FGF1 also induced phosphorylation of PLCγ1, an FRS2-independent component of the FGFR pathway, which was completely abolished by the treatment with either FGFR inhibitor. The expression and phosphorylation of AKT was unaffected by FGF1 stimulation or FGFR inhibition (Figure 4A). Thus, no clear differences in FGF signaling could be seen that explained the difference in growth inhibition of the two inhibitors in NRH-LS1.

We further compared the effect of LY2874455 on the same proteins on the three cell lines to understand their difference in sensitivity to FGFR inhibition (Figure 4B, quantified in Figure S4B). The western blots confirmed that FRS2 protein was expressed in all three cell lines, while pFRS2 was below detection in unstimulated cells. Phosphorylation of FRS2, PLCγ1, and ERK was induced by stimulation with FGF1 and was inhibited upon treatment with LY2874455 in all three cell lines, showing the ability of these cell lines to respond to FGF stimulation and FGFR inhibition. The phosphorylation levels of AKT were higher in both LPS510 and LPS853 compared to NRH-LS1 but remained unaffected by stimulation or inhibition of FGFR signaling (Figure 4B). 

### 3.5. The Expression of FGF Receptors Varies in FRS2-Amplified LPS Lines

In order to investigate why only two out of three LPS cell lines responded to the treatment with FGFR inhibitors and whether this could be explained due to a differential expression of some components of the FGFR pathways, we analyzed transcriptome sequencing data of NRH-LS1, LPS510, and LPS853 for expression of FGFR signaling components upstream of FRS2. Similar relative expression (FPKM) values of 52.0, 51.8 and 44.3 for *FRS2* were found in NRH-LS1, LPS510 and LPS853, respectively. All three cell lines also had similar expression levels for *FGFR1* with FPKMs of 41.5, 51.5 and 36.1, while *FGFR4* was comparably higher expressed in LPS853 with an FPKM of 77.5, compared to 1.2 and 0 in NRH-LS1 and LPS510, respectively. All the cell lines had very low expression of *FGFR2* and *FGFR3*. *FGFRL1* is another member of the fibroblast growth factor receptor family, however it lacks the cytoplasmic tyrosine kinase domain and can act as a negative regulator of FGFR signaling [18]. Interestingly, *FGFRL1* was higher expressed in LPS853 than in LPS510 and NRH-LS1, with FPKM values of 50.0, 25.8 and 11.1, respectively.

We also found *FGF2* to be higher expressed in NRH-LS1 and LPS510 with FPKM values of 19.6 and 21.0 respectively, compared to LPS853 with FPKM of 0.5. In turn, *FGF11* expression was higher in LPS853 with FPKM of 24.5, compared to NRH-LS1 and LPS510 with values of 0.4 and 1.2 respectively. A heat map that shows the expression of FGF receptors and ligands, as well as adapter proteins, is provided in Figure S3.

### 3.6. LY2874455 Inhibits Tumor Growth In Vivo

**Figure 5:**
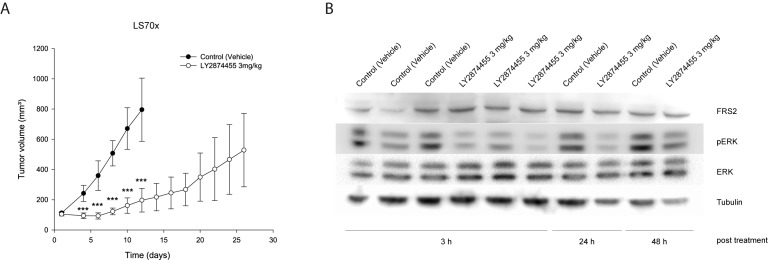
Growth inhibitory effect of LY2874455 *in vivo* on LS70x, a *FRS2*-amplified xenograft. (**A**) *in vivo* study with *FRS2*-amplified tumors of LS70x xenografts (*n* = 6) treated twice per day (BID) with LY2874455 3 mg/kg or control vehicle for up to 28 days until tumor size reaches a limit of 1 cm^3^. Data shown as means ± SEM *** *p* ≤ 0.001; unpaired two-tailed *t*-test treated versus vehicle treated; (**B**) Western blots showing the level of phosphorylated and total ERK in lysates extracted from LS70x tumors treated *in vivo* with vehicle or LY2874455 until endpoint. The tumors were harvested at the indicated time after last treatment. α-tubulin is used as loading control.

Having observed promising therapeutic potential *in vitro* for two out of three *FRS2*-amplified LPS cell lines treated with LY2874455, we investigated the effect *in vivo*. Patient-derived xenograft (PDX) models derived directly from patient material are more representative for drug responses, but the NRH-LS1 PDX model, from which the cell line is derived, grows slowly and was not suitable for preclinical testing. Rather than making a less representative cell line-derived PDX from LPS510, we used the DDLPS patient-derived LS70x PDX [14], which also provided an additional independent model. LS70x has amplification and increased expression of *FRS2* comparable to the three cell lines (Figure S1), and grows reasonably well in NSG mice. When tumors reached 150 mm^3^, mice were treated with 3 mg/kg twice per day. Already from day 4 of treatment we observed significant inhibition of tumor growth compared to control-treated mice (Figure 5A). To confirm that LY2874455 reduced FGFR signaling *in vivo*, we performed a kinetic study of FGFR signaling proteins. Tumors were harvested at 3, 24 and 48 h after last treatment (end of study) and protein lysates were subjected to Western blotting to analyze the phosphorylation levels of FRS2 and ERK. The endogenous level of phosphorylated FRS2 in the LS70x tumors was, as in the cell lines, below detection (data not shown). However, ERK phosphorylation was clearly reduced 3 h after treatment and remained reduced for at least 24 h (Figure 5B, quantified in Figure S4C).

## 4. Discussion

WD/DDLPS tumors almost invariably have *FRS2* amplified [8] and are in great need of new therapies. We cannot expect many new drugs specific for rare cancers, therefore the possibility to repurpose existing drugs for these patients deserves thorough consideration. FGF receptor inhibitors have shown tolerable toxicities in rodent and patients [10,11], thus showing efficacy in preclinical models could pave the way to clinical trials on sarcoma patients.

Our previous *in vitro* study demonstrated limitations of NVP-BGJ398, since the drug was only transiently cytostatic and the cells quickly regained growth capacity when drug was removed [3]. In this study we further investigated the potential of FGFR inhibition as treatment for *FRS2*-amplified DDLPS.

Results were more promising using the FGFR inhibitor LY2874455, which gave improved efficacy *in vitro*. LY2874455 is reported to have a similar potency against all four FGFRs in biochemical assays and has shown potent activity against FGFR signaling in preclinical studies of several cancer types such as lung, gastric, bladder and multiple myeloma [12]. This drug had a stronger effect on the growth of NRH-LS1 cells, and induced apoptosis more efficiently than NVP-BGJ398 did. Interestingly, LY2874455 had a long-lasting effect on the responding cell lines, with cell growth inhibited several days after the drug treatment was discontinued. The observed effect and higher potency of LY2874455 compared to NVP-BGJ398 could potentially be due to off-target inhibition, since LY2874455 was shown to act as a multi-kinase inhibitor and consequently inhibits a wide range of different kinases [19]. However, the similarity of the response of FGFR signaling by the two inhibitors indicates a similar mode of action.

We hypothesized that amplified *FRS2* would potentiate FGFR signaling and drive growth of *FRS2*-amplified DDLPS. Thus, *FRS2* amplification could be a biomarker predicting sensitivity to FGFR inhibitors. Although the three *FRS2* amplified cell lines had similar levels of *FRS2*, LPS853 was resistant, suggesting that *FRS2* amplification alone is not sufficient as predictive biomarker. The lack of response of one cell line out of four independent models might not be representative of the patient population, but more refined biomarkers detecting such tumors would be valuable. To identify possible differences in FGFR signaling that could explain the different responses to FGFR inhibition, we analyzed the status of FGFR signaling pathway proteins. The basal level of phosphorylated FRS2 in unstimulated cells was undetectable but was drastically increased upon addition of FGF1 for all the three cell lines, confirming functional FGFR signaling. Both AKT and ERK are downstream of FRS2 and the strong inhibition of ERK phosphorylation when cells were treated with FGFR inhibitor indicated that the FGFR pathway is the predominant activator of ERK in these cells. This is consistent with a study in human bladder cancer, which showed dephosphorylation of FRS2 and ERK by NVP-BGJ398 [10]. We could not detect significant changes in levels of AKT or phosphorylated AKT, which is also consistent with previous studies [20].

We did not observe any FGFR mutations or translocations that could explain the different response to FGFR inhibition (data not shown). Although we observed equal expression of *FGFR1* among the cell lines, the non-responding LPS853 cells had considerably higher expression of *FGFR4*. Furthermore, LPS853 also had a high expression of *FGFRL1*, which lacks the kinase domain and has been suggested to be a negative regulator of FGFR1 signaling [21]. Although LPS853 cells do not respond to FGF inhibitors with reduced proliferation, FGFR inhibition still prevents endogenous and FGF1-induced ERK phosphorylation in these cells. This implies that FGFR signaling is maintained in LPS853 cells, but that these cells are not dependent on phosphorylated ERK for proliferation. This is known for other cell types as well. Only a subset of KRAS mutated colon, pancreatic and lung cancer depends on MEK/ERK signaling for proliferation, although the cells responds to MEK inhibition with reduced pERK [22]. Often this is due to other rescuing mechanisms. This could explain why LPS853 cells grow independent of this pathway, and also why they respond to higher doses of LY2874455, but not to high doses of NVP-BGJ398 (data not shown) [3], since LY2874455, unlike NVP-BGJ398, has a similar potency against all four FGF receptors in biochemical assays [12,23].

We expect the levels of FGFs in fetal calf serum to be low, but the responding cell lines may produce autocrine FGFs, as was indeed indicated by the RNA-seq data. NRH-LS1 and LPS510 had high expression of *FGF2*, a ligand for several of the FGF receptors, while LPS853 had high expression of the intracellular FGF11. While the function of FGF11 is not fully known, FGF2 is known to be secreted by adipocytes and to stimulate proliferation upon binding to FGFR1. We hypothesize that the lack of expression of any extracellular FGF ligands in LPS853 cells indicate that they grow independently of FGFR *in vitro*, but we cannot exclude that they depended on exogeneous FGF *in vivo* and have adapted to conditions without FGF *in vitro*. Further functional investigations including knock-down of the different ligands and receptors might solve these issues.

In summary, our results support LY2874455 as a better drug candidate than NVP-BGJ398 for treatment of *FRS2*-amplified liposarcoma. LY2874455 also showed significant efficacy *in vivo*, which is an important finding, since the FGF-regulatory landscape in tissues is different from cell cultures. Whether efficacy of LY2874455 can also be translated to other sarcomas with aberrations in the FGFR pathway, such as amplified, fused or mutated *FGFR* genes [24], needs to be investigated. We hope these studies could result in a clinical trial for DDLPS patients in great need of new treatments now that phase I clinical trials have shown tolerable toxicities [11].

## Supplementary Materials

The following are available online at https://www.mdpi.com/2073-4409/8/2/189/s1, Figures S1–S4.
