# Supplementary material for: Preclinical Evaluation of the Pan-FGFR Inhibitor LY2874455 in FRS2-Amplified Liposarcoma
Source: Cells. 2019 Feb 21;8(2):189. doi: 10.3390/cells8020189 (PMC6406403; doi:10.3390/cells8020189)
Supplement: Supplementary file 1 [file cells-08-00189-s001.pdf]

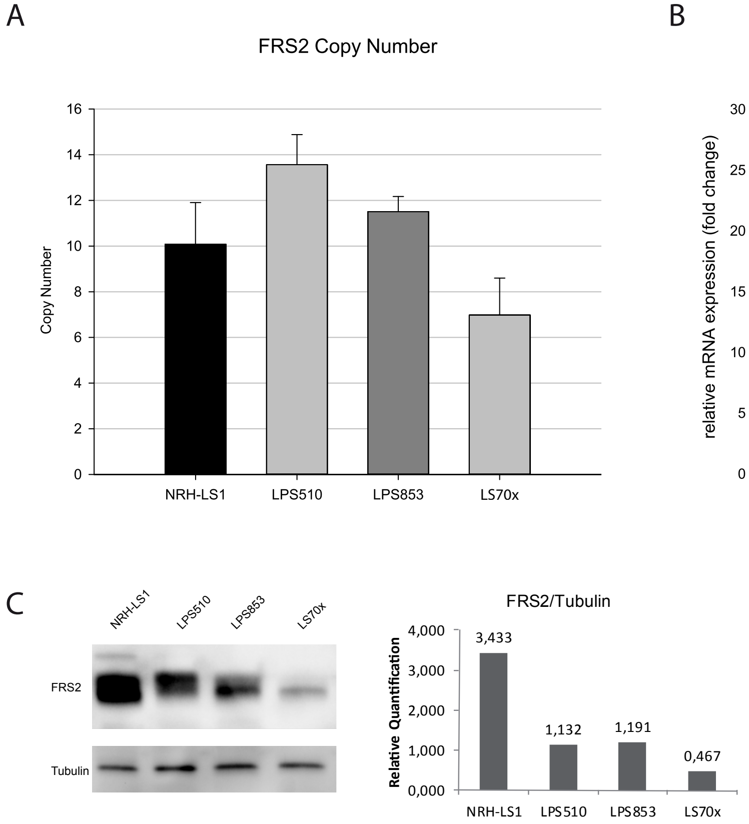

Supplementary Figure S1: FRS2 Copy Number and Gene Expression Analysis in NRH-LS1, LPS510 and LPS853 cells, and LS70x xenograft. **A,** The number of FRS2 gene copies relative to LSAMP; one representative experiment is shown (n=3), error bars represent standard deviation (SD) of technical replicates. **B,** Relative gene expression of FRS2 compared to B2M relative to human adipose tissue; error bars represent standard deviation (SD) of experiments (n=2, iMSC#3 n=1). **C,** Western blot with quantification showing the total FRS2 levels in the three cell lines and the LS70x xenograft.

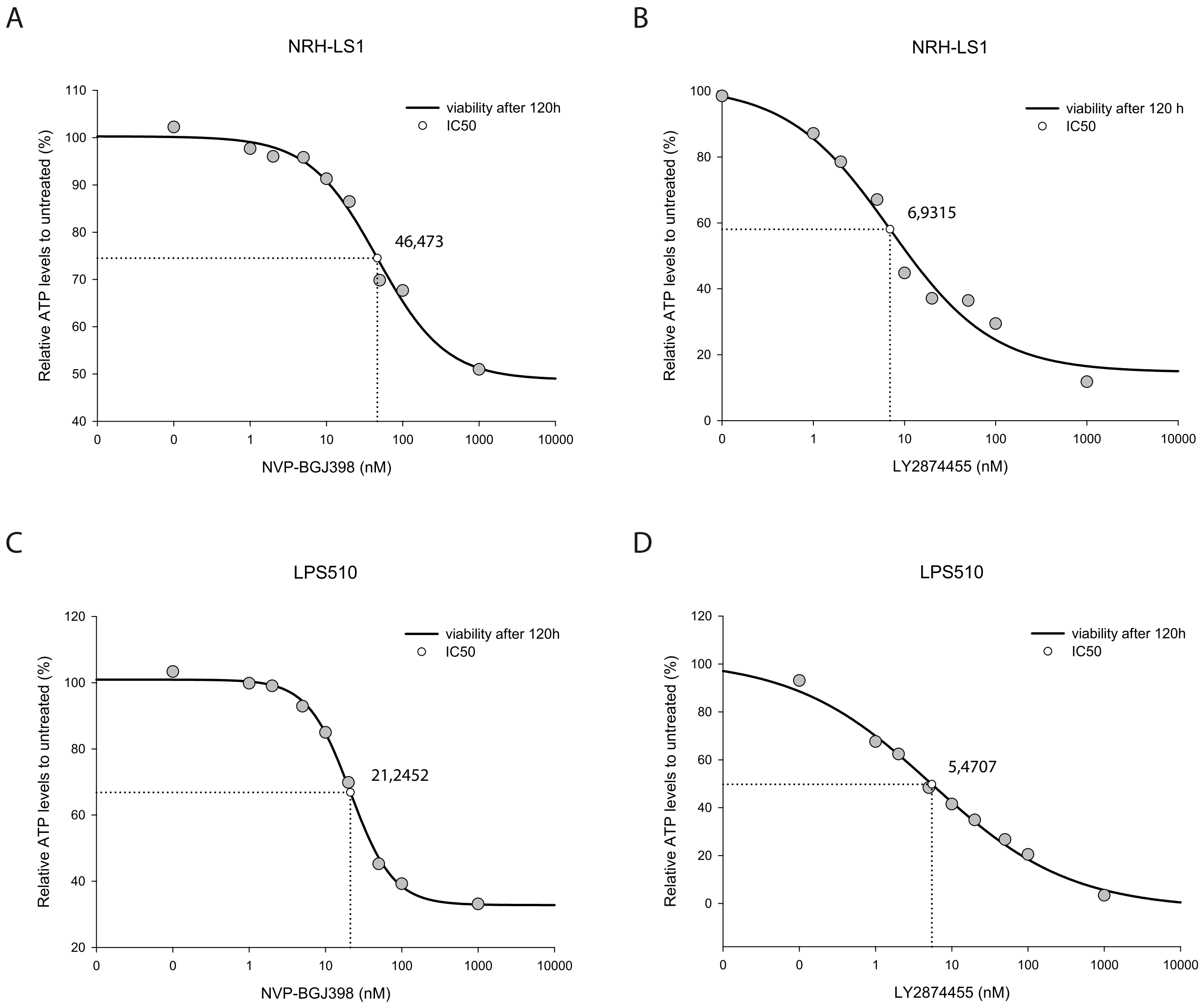

Supplementary Figure S2: LY2874455 has better efficacy than NVP-BGJ398. A, The IC50 was estimated for NRH-LS1 treated with NVP-BGJ398 at 46 nM. **B,** The IC50 was estimated for NRH-LS1 treated with LY2874455 at 7 nM. **C,** The IC50 was estimated for LPS510 treated with NVP-BGJ398 at 21 nM. **D,** The IC50 was estimated for LPS510 treated with LY2874455 at 5 nM. **A-D,** Cell viability measured by relative ATP levels to untreated after 120h of treatment.

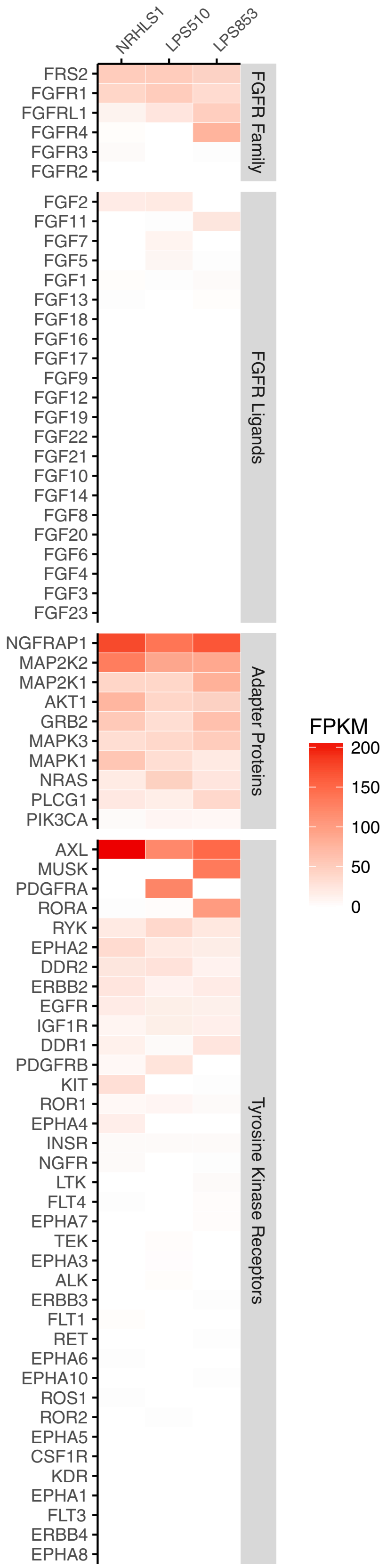

Supplementary Figure S3: Heatmap of expression of FGFR pathway genes and other tyrosine kinases between NRH-LS1, LSP510 and LPS853 cell lines. Relative expression levels (FKPMs) for each gene is presented in separate panels showing FGFRs and FRS2 (top panel), FGF ligands, adapter proteins downstream in the FGF pathway (except for NGFRAP1), and other tyrosine kinase receptors. Genes are sorted by FPKM values in each panel.

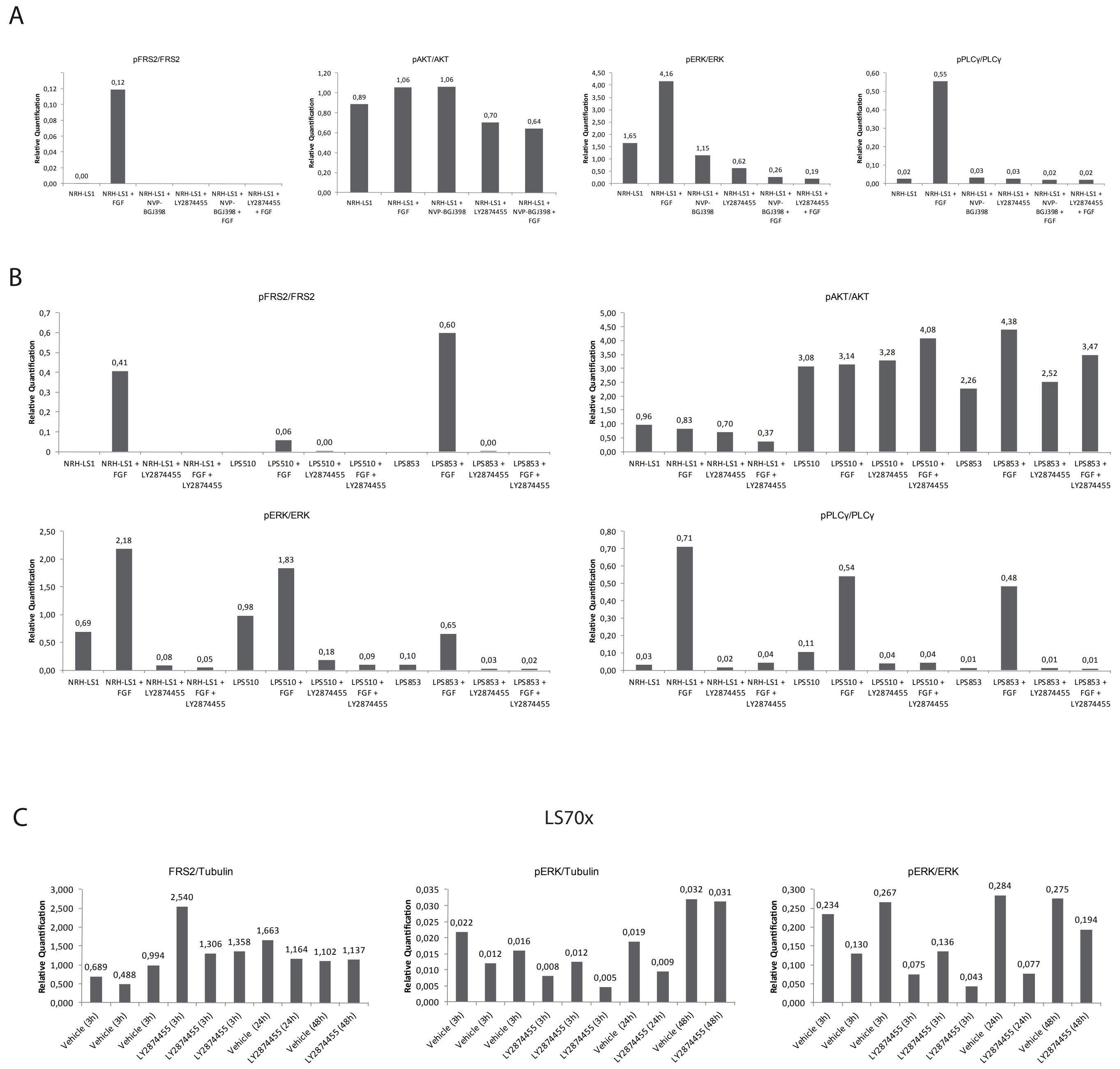

Supplementary Figure S4: Quantifications of western blots from Figure 4A (A), Figure 4B (B), and Figure 5B (C).
